# A Pharmacokinetic Study of Antimalarial 3,5-Diaryl-2-aminopyridine Derivatives

**DOI:** 10.1155/2015/405962

**Published:** 2015-03-29

**Authors:** Ntokozo Dambuza, Peter Smith, Alicia Evans, Dale Taylor, Kelly Chibale, Lubbe Wiesner

**Affiliations:** ^1^Division of Clinical Pharmacology, Department of Medicine, University of Cape Town, Observatory, Cape Town 7925, South Africa; ^2^Department of Chemistry, University of Cape Town, Rondebosch, Cape Town 7700, South Africa

## Abstract

Malaria caused by *Plasmodium falciparum* is responsible for approximately 80% of the incidence and 90% of deaths which occur in the World Health Organization (WHO) African region, with children and pregnant women having the highest incidence. *P. falciparum* has developed resistance, and therefore new effective candidate antimalarial drugs need to be developed. Previous studies identified 3,5-diaryl-2-aminopyridines as potential antimalarial drug candidates; therefore, derivatives of these compounds were synthesized in order to improve their desired properties and pharmacokinetic (PK) properties of the derivatives were investigated in a mouse model which was dosed orally and intravenously. Collected blood samples were analyzed using liquid chromatography coupled to mass spectrometer (LC-MS/MS). The mean peak plasma level of 1.9 *μ*M was obtained at 1 hour for compound 1 and 3.3 *μ*M at 0.5 hours for compound 2. A decline in concentration was observed with a half-life of 2.53 and 0.87 hours for compound 1 in mice dosed orally and intravenously, respectively. For compound 2 a half-life of 2.96 and 0.68 hours was recorded. The bioavailability was 69% and 59.7% for compound 1 and compound 2, respectively.

## 1. Introduction

Malaria is a disease caused by parasites from the genus* Plasmodium* and is transmitted to a human host when a vector, an infected female* Anopheles* mosquito, bites the host [1].* P. falciparum *and* P. vivax* are responsible for most malaria infections, which has become one of the major public health problems in many regions of Africa, Asia, and Latin America [2]. The World Health Organization (WHO) estimated 198 million cases of malaria and 584 000 deaths occurred globally in 2013 with the most occurrence in Africa. Most deaths (78%) were in children under the age of five [3]. Resistance to antimalarials has been reported in both* P. falciparum *and* P. vivax*, but their resistance pattern differs and requires the development of new candidates of effective antimalarial drugs [4].

Recent studies by Gonzalez Cabrera et al. [5] and Younis et al. [6, 7] identified 3,5-diaryl-2-aminopyridines as potential antimalarial drug candidates based on* in vivo* efficacy data obtained from a* P. berghei *infected mouse model. These compounds were identified from an image-based high-throughput screening of a BioFocus DPI SoftFocus kinase library of promising selective* in vitro* antiplasmodial hits. A number of active compounds with varying aryl groups at positions 3 and 5 of the aminopyridine core were identified and analogues were synthesized in order to explore the structure-activity relationship (SAR) around aryl substitution. Some of these analogues showed good oral bioavailability in a pharmacokinetic study performed in rats, identifying them as potential clinical candidates [6]. This led to the synthesis of new derivatives of 3,5-diaryl-2-aminopyridines with the aim of overcoming liabilities such as the human Ether-à-go-go-Related Gene (hERG) activity, which was identified in an earlier study as a property that is not ideal for a clinical candidate [5, 6].

**Figure 1:**
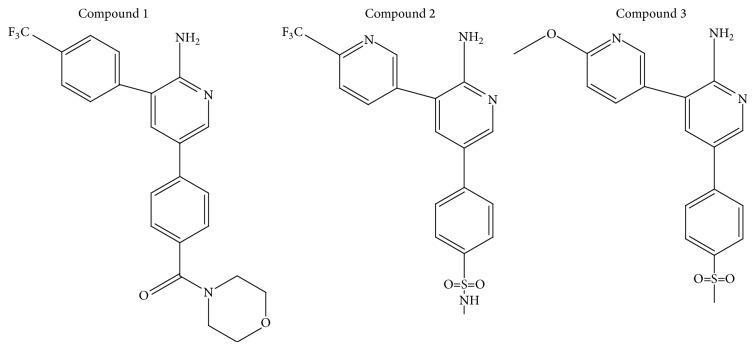
The structures of synthesized 3,5-diaryl-2-aminopyridines derivatives (compounds 1 and 2) and their parent compound 3.

Compounds 1 and 2 (Figure 1) are derivatives of compound 3, an active 3,5-diaryl-2-aminopyridine antimalarial (Figure 1). Studies conducted by Gonzalez Cabrera et al. [5] presented data that showed the potential antimalarial activity of these derivatives when tested* in vitro *against chloroquine-sensitive and chloroquine-resistant* P. falciparum *strains displayed activity in a* P. berghei* infected mouse model. These compounds also showed metabolic stability and reduced hERG activity compared to the parent, that is, compound 3. Therefore, this study aims to investigate the pharmacokinetic properties of these derivatives.

## 2. Materials and Methods

### 2.1. Reagents

Dimethyl sulfoxide (Merck), phosphate buffered saline tablets (Sigma), and heparin-coated MiniCollect Plasma Tubes (Lasec SA) were obtained. All other solvents and chemicals used were of an HPLC and an analytical grade.

### 2.2. Experimental Animals

Male and female C57BL/6 mice (20–30 g) were obtained from the University of Cape Town Medical School Animal Unit. The mice were housed in ventilated cages at room temperature (approximately 22°C) with a constant supply of food and water and were monitored twice daily. The study was authorized by the University of Cape Town's Faculty of Health Science Animal Research Ethics Committee before its commencement (Reference number 012/020). All the work was performed according to the guidelines established by Austin and colleagues [8].

### 2.3. Test Sample Preparation

The compounds (Figure 1) were dissolved in DMSO and diluted further with phosphate buffered saline (PBS) to give a final DMSO concentration of 1%.

### 2.4. Animal Protocol

Comprehensive PK studies were performed on groups of five animals to determine the pharmacokinetic properties of the test compounds. This involved the oral dosing (200 *μ*L) of 10-week-old C57BL/6 male mice at 20 mg/kg and intravenous dosing (100 *μ*L) under anesthesia at 4 mg/kg via the dorsal penile vein. For the oral dosage a gavage needle was used for the administration of test compounds directly into the lower esophagus or stomach. Blood samples (approximately 30 *μ*L) were collected serially by needle prick on the tail vein near to the tip of the tail at 0, 0.17, 0.5, 1, 2, 3, 5, 7, and 9 hours after dosing. Lithium-heparin-coated tubes were used to collect the blood samples. The collected blood samples were placed on ice immediately after sampling and were frozen at −80°C until analyzed.

### 2.5. Blood Sample Preparation

The blood samples were brought to room temperature and then mixed by vortexing to ensure homogeneity. Twenty microliters of blood was mixed with 50 *μ*L Milli-Q water and 150 *μ*L acetonitrile. The sample was vortexed for 15 seconds, sonicated for 10 minutes, and centrifuged at 13000 ×g for 5 minutes. The supernatant was transferred to a flat-bottom glass insert and placed in a glass vial for analysis.

### 2.6. Chromatography

The liquid chromatography (LC) system employed was an ultrafast liquid chromatography (UFLC) system (Shimadzu) and the separation of the compounds was performed on a Phenomenex, Luna 5 *μ*m PFP(2), 100 Å, and 50 mm × 2 mm analytical column. For method development, the compounds were prepared at 1 mg/mL stock in methanol and were further diluted with 50% acetonitrile: 0.1% formic acid to make a final concentration of 1 *μ*g/mL. Analyst software version 1.5.2 (AB Sciex) was employed for data acquisition, peak-area integration, and quantitation of compounds in blood samples.

The mobile phase A consisted of 0.1% formic acid in water (v/v) and mobile phase B consisted of acetonitrile. Samples were maintained at 4°C until injection. The flow rate was set at 500 *μ*L/min and the temperature of the column was maintained at 40°C. For the separation of the compounds the mobile phase was increased from 5% to 95% B over 2 minutes and returned to 5% B within 0.1 minutes and then equilibrated for 3 minutes. Calibration curves were derived in each analytical run in duplicate and were employed to extrapolate the concentration of the compounds in the blood samples.

### 2.7. Detection

The MS system consisted of an AB Sciex 3200 Q-Trap mass spectrometer which was operated at unit resolution in the multiple reaction monitoring (MRM) mode. The calibration range for all the compounds was between 7.8 and 1000 ng/mL and the accuracy (%Nom) for the calibration curves was between 90.31 and 104.0%.  Table 1 gives an overview of the MS parameters and the instrument settings.

### 2.8. Pharmacokinetic Sample Analysis

Noncompartmental analysis was performed on each individual set of data using PK Solutions 2.0 Pharmacokinetic Analysis Software (Summit Research Services, Montrose, USA), which uses an automated Excel-based program. The following PK parameters were calculated using PK solutions equations listed in program (http://www.summitpk.com/equations/equations.htm): maximum blood concentration (*C*_*Max*⁡_ [*μ*M]) and corresponding time (*T*_*Max*⁡_ [min]), apparent terminal half-life (*t*_1/2_ [min]), total exposure (AUC_0–*∞*_ [*μ*M·min]), volume of distribution (Vss [L/kg]), blood clearance (CL [L/min/kg]), and percentage oral bioavailability (%BA).

## 3. Results

**Figure 2:**
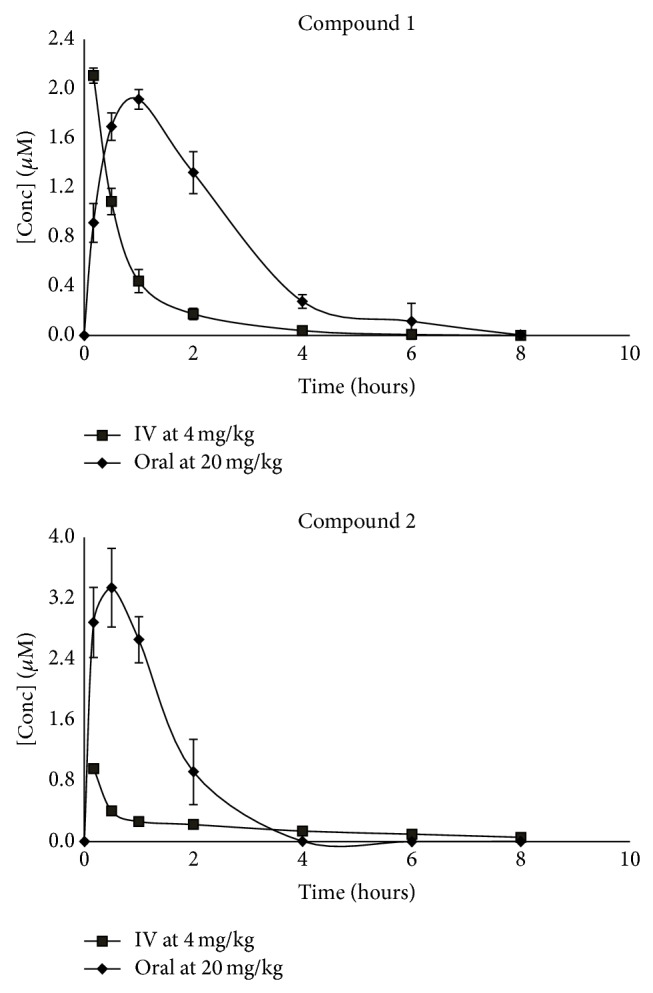
Blood concentrations of compounds 1 and 2 in C57BL/6 mice blood after oral and intravenous administration. Data represent mean ± standard deviation of data points obtained from five single mice.

A pharmacokinetic study was performed on two 2-aminopyridine compounds, namely, compounds 1 and 2. Five mice were used for each route of administration, where each compound was dosed at 20 mg/kg orally and 4 mg/kg intravenously (IV). The mean concentration versus time profiles of compounds 1 and 2 obtained after the mice were dosed is given in Figure 2. The pharmacokinetic parameters for the oral and IV groups are presented in Table 2.

After administering 20 mg/kg of compound 1 orally, a maximum concentration of 1.915 ± 0.03 *μ*M was reached within 1 hour with a moderate terminal half-life of 2.53 ± 0.1 hours, which was longer than the IV half-life of 0.87 ± 0.03 hours. The oral AUC_0–*∞*_ was 359.3 ± 97 and the IV AUC was 103.4 ± 11 *μ*M·min, respectively. A high volume of distribution of 9.51 ± 1 L/kg and a high clearance rate of 83.6 ± 12 mL/min/kg were observed. The oral bioavailability of compound 1 was 69 ± 19%.

Compound 2 reached a maximum concentration of 3.34 ± 0.2 *μ*M within 30 minutes and had moderate terminal half-life of 2.96 ± 0.2 hours, which was longer than a half-life of 0.68 ± 0.6 hours for the intravenous dose. AUC_0–*∞*_ for the mice dosed orally was 313.6 ± 30 and 105.1 ± 23 *μ*M·min for mice dosed intravenously. The volume of distribution was very high at 27 ± 1.3 L/kg with a high clearance rate of 92.6 ± 19 mL/min/kg. Compound 2 had a moderate oral bioavailability of 60 ± 5%.

## 4. Discussion and Conclusion

The two compounds evaluated in this study are potent antimalarials derived from 3,5-diaryl-2-aminopyridine (compound 3) that was identified as a potential clinical trial candidate for antimalarial treatment in a previous study [5, 6]. Compounds 1 and 2 were synthesized in order to minimise the liabilities such as hERG activity associated with compound 3. The activity of the derivatives was evaluated in another study and improved IC_50_ values were observed in the range from 7 to 14 nM for* P. falciparum* chloroquine-sensitive strains (D10 and 3D7) and chloroquine-resistant strains (Dd2 and K1) when comparing to the parent compound 3 with IC50 values in the range from 16 to 194 nM [6]. This study was designed, therefore, to evaluate the pharmacokinetic properties of these two derivatives in a mouse model.

Both compounds 1 and 2 were absorbed and distributed into various tissues quickly following an oral dose. Data in Table 2 showed a rapid absorption with maximum concentrations reached within 1 hour after drug administration. The high volume of distribution and a high clearance rate may have influenced the short half-life of these compounds, which range between 2.5 and 3 hours for mice dosed orally and between 0.68 and 0.87 hours for mice dosed intravenously.

Even though the bioavailability of compounds 1 and 2 was high, it is a parameter that determines the amount of compound reaching the systemic circulation, which in turn may affect the pharmacological response [9]. Other pharmacokinetic parameters were affected significantly. These parameters include an increased clearance rate and a significant reduction in their half-life. A pharmacokinetic study of compound 3 was performed on male Sprague Dawley rats with a clearance rate of 18.3 mL/min/kg and apparent *t*_1/2_ values of 6.5 and 8 hours for rats dosed orally and intravenously, respectively. The oral and intravenous AUC values of compound 3 were 6880 and 789 *μ*M·min, respectively, with an oral bioavailability of >100% [6]. Unfortunately, the PK parameters presented in this study for compounds 1 and 2 could not be compared to those of the parent compound 3 because of the physiological differences between the two species [10]. Therefore, the data presented in this study can provide a baseline for further exploration of more derivatives in order to optimize the pharmacokinetic properties while retaining high potency* in vitro *and metabolic stability.

## Figures and Tables

**Table 1:** Mass spectrometer settings and MS parameters used for the detection of the test compounds on an API 3200 Q-Trap.

Parameter	Compound 1	Compound 2
Q1 mass (Da)	428.1	408.9
Q3 mass (Da)	227.2	315.2
Dwell time (msec)	40	40
Declustering potential	81	101
Collision energy (Volts)	77	55
Entrance potential (Volts)	10	10
Collision cell exit potential (Volts)	4	2
Source temperature	500°C	500°C
Curtain gas (psi)	25	25
Gas 1 (psi)	50	50
Gas 2 (psi)	70	70
CAD gas	Medium	Medium
Ion spray voltage (kVolts)	5500	5500
Ionization mode	Positive	Positive

**Table 2:** Pharmacokinetic parameters of 3,5-diaryl-2-aminopyridine compounds after oral and intravenous administration. Data represent mean ± standard deviation of data points obtained from five mice.

Parameters	Compound 1		Compound 2	
	Oral	IV	Oral	IV
*T*_1/2_ (hours)	2.53 ± 0.1	0.87 ± 0.03	2.96 ± 0.2	0.68 ± 0.6
*T*_*Max*⁡_ (hours)	1	NA^*^	0.5	NA
*C*_*Max*⁡_ (*μ*M)	1.915 ± 0.03	2.11 ± 0.06	3.337 ± 0.2	0.96 ± 0.06
Vss (L/kg)	ND^*^	9.51 ± 1	ND	27 ± 1.3
CL (mL/min/kg)	ND	83.6 ± 12	ND	92.6 ± 19
AUC_0–*∞*_ (*µ*M·min)	359.3 ± 97	103.4 ± 11	313.6 ± 30	105.1 ± 23
BA (%)	69 ± 19	NA	60 ± 5	NA

^∗^ND indicates that the value was not determined. NA: not applicable.
